# Navigating climate change: A qualitative study on mental health and behavioral changes among older adults

**DOI:** 10.1192/j.eurpsy.2026.11102

**Published:** 2026-07-31

**Authors:** S. von Humboldt, N. Ilyas, I. Leal

**Affiliations:** 1 William James Center for Research, ISPA – Instituto Universitário, Lisbon, Portugal; 2 Center for Clinical Psychology, University of the Punjab, Lahore, Pakistan

## Abstract

**Introduction:**

The effects of climate change increasingly shape the mental health and well-being of older adults.

**Objectives:**

This study aims to (1) explore older adults’ experiences related to climate change and (2) analyze its influence on their mental health and self-reported behavioral changes.

**Methods:**

A qualitative study involving 578 older adults was conducted, utilizing in-depth interviews to gather data.

**Results:**

Content analysis revealed five main themes for the first objective: (1) Sudden health complications (89.6%), (2) Heightened reliance on healthcare providers (77.2%), (3) Greater burden on family members (75.6%), (4) Challenges in accessing relief and services (62.1%), and (5) Elevated risk of falls (57.8%). Regarding mental health, three themes emerged: (1) Feelings of distress and anxiety (88.1%), (2) Experiences of emotional trauma (79.4%), and (3) Episodes of emotional outbursts (55.2%). Finally, the following changes of behavior were indicated: (1) Disrupted sleep patterns (58.7%), (2) Increased aggression (42.9%), and (3) Poor dietary habits (41.3%).

**Conclusions:**

The findings underscore the critical role of improving healthcare services alongside comprehensive support strategies to alleviate mental health and behavioral difficulties faced by older adults amid climate change impacts.

Keywords: Changes in behavior; climate change; mental health; older adults; qualitative study.

**Disclosure of Interest:**

None Declared

